# Intermittent Administration Alleviates Enrofloxacin-Induced Hepatotoxicity in Zebrafish via Adaptive Lipid Remodeling and p38 MAPK Signaling

**DOI:** 10.3390/biology15161354

**Published:** 2026-08-10

**Authors:** Jiangkun Yu, Yiming Liu, Yake Gao, Dingxi Pan, Mingying Li, Wei Guo

**Affiliations:** 1 State Key Laboratory of Conservation and Utilization of Bio-Resources, School of Life Sciences, Center for Life Sciences, Yunnan University, Kunming 650500, China; 2 School of Ecology and Environmental Science, Yunnan University, Kunming 650500, China

**Keywords:** enrofloxacin, administration regimen, MAPK, lipid metabolism, zebrafish

## Abstract

Enrofloxacin (ENR) has been widely used in global aquaculture production, yet its hepatotoxic impacts under different operational administration modes remain poorly understood. Current environmental risk assessments and therapeutic guidelines generally assume that cumulative exposure dose dictates biological outcomes, largely ignoring the temporal patterns of drug administration. In the present study, we systematically compared the hepatotoxic, metabolic, and molecular effects of two matched-duration ENR administration regimens, continuous versus intermittent, on adult zebrafish (*Danio rerio*) at environmentally realistic concentrations (0.4, 4, and 40 μg/L). Biochemical, histological, and lipidomic analyses revealed fundamentally divergent toxicological trajectories between the two regimens. While both regimens induced significant basal triglyceride and cholesterol depletion, continuous exposure drove progressive hepatocellular damage, characterized by elevated transaminase activities, unconstrained glycogen accumulation, and the eventual collapse of antioxidant defenses, culminating in severe lipid peroxidation. Mechanistically, continuous exposure triggered sustained pathological activation of the p38 MAPK signaling cascade and broad upregulation of de novo phosphatidylcholine (PC) and phosphatidylethanolamine (PE) biosynthesis. In contrast, intermittent exposure provided a critical physiological buffering window, whereby the depuration phase enabled a sustained, adaptive antioxidant response that successfully suppressed lipid peroxidation, significantly alleviated structural liver injury, and raised the activation threshold for the p38 stress response. Furthermore, lipidomic and transcriptional profiling demonstrated that intermittent exposure uniquely induced compensatory lipid remodeling, characterized by adaptive ceramide signaling and a metabolic shift. Taken together, these findings demonstrate that the temporal pattern of exposure critically dictates ENR hepatotoxicity, highlighting the need to perform discriminative risk assessment and providing a mechanistic basis for optimizing antibiotic stewardship in aquaculture.

## Simple Summary

How does antibiotic administration affect the health of farmed fish? In this study, zebrafish were exposed to enrofloxacin—a common aquaculture antibiotic—to evaluate physiological, histological, and metabolic changes in the liver using either a continuous or an intermittent regimen. We found that continuous exposure caused severe liver damage and biological stress. However, giving the fish a short break (intermittent exposure) dramatically protected their livers. This break allowed the fish to adapt and heal, preventing cellular damage despite receiving the same total amount of medication. These findings suggest that adopting “pulsed” dosing could significantly improve animal welfare and promote sustainable fish farming.

## 1. Introduction

Aquaculture represents the world’s fastest-growing food production sector, yet its intensive reliance on antibiotics has rendered aquatic environments a major reservoir of pharmaceutical residues [1,2]. Fluoroquinolones, in particular, have been widely detected at concentrations ranging from ng/L to μg/L in surface waters and aquaculture effluents, reflecting their ubiquitous distribution across freshwater and coastal systems [3,4,5]. Among these compounds, enrofloxacin (ENR) constitutes the most heavily used antibiotic in global aquaculture production and the most frequently detected residue in farmed fish [6,7]. Although ENR demonstrates potent bactericidal efficacy, a growing body of evidence has indicated that even environmentally realistic concentrations disrupt physiological, behavioral, and immune functions in fish, thereby threatening individual health and population stability [8,9].

The teleost liver serves as the central organ for xenobiotic metabolism and detoxification, coordinating lipid metabolism, energy homeostasis, and immune defense in response to environmental challenges [10,11]. Notably, ENR preferentially accumulates in hepatic tissue and undergoes biotransformation into ciprofloxacin via hepatic cytochrome P450 enzymes, a process that further amplifies the toxicological burden imposed on the liver [12,13]. At the molecular level, ENR and its metabolites disrupt the delicate balance between reactive oxygen species (ROS) generation and antioxidant defenses, leading to oxidative stress, lipid peroxidation, and altered energy metabolism [14,15]. Sustained oxidative stress ultimately causes hepatocellular damage, manifested as elevated alanine aminotransferase and aspartate aminotransferase activities, depletion of hepatic lipid reserves, and pronounced histopathological alterations [16,17].

To date, toxicological studies concerning ENR hepatotoxicity in fish have predominantly focused on determining the threshold concentrations that elicit measurable injury and on evaluating the persistence of damage following long-term, continuous exposure. These investigations have established clear dose–response profiles and well-characterized injury paradigms under sustained chemical stress [9,18,19,20]. However, the prevailing experimental models often diverge from practical aquaculture applications, where producers face a critical operational dichotomy regarding drug administration that bears distinct biological consequences. In some instances, treatment courses are deliberately extended beyond labeled durations to ensure therapeutic efficacy against persistent infections, effectively resulting in continuous exposure at sub-lethal concentrations. Conversely, producers may employ intermittent administration regimens, interspersing therapeutic pulses with drug-free recovery periods to mitigate hepatic and microbial burdens while maintaining disease control. Whether this intermittent administration alleviates or exacerbates hepatotoxicity compared with a matched-duration continuous regimen remains poorly understood, warranting more research.

To bridge this gap, the present study exposed adult zebrafish (*Danio rerio*) to systematically compare the hepatotoxic effects of two matched-duration ENR administration modes, continuous and intermittent, under environmentally realistic concentrations (0.4, 4, and 40 μg/L) referenced to commercial therapeutic doses and previous experimental work [14,16]. By integrating hepatic lipidomic profiling with conventional biochemical assays, antioxidant capacity evaluations, and histopathology, we aimed to elucidate the distinct impacts of these regimens. The continuous regimen simulates the operational extension of treatments for sustained therapeutic coverage, whereas the intermittent regimen models the cyclical exposure strategy employed to reduce hepatic strain. The findings potentially enhance the current understanding of how temporal exposure patterns modulate chemical hepatotoxicity and facilitate regimen-guided discriminative risk assessment in aquaculture.

## 2. Materials and Methods

### 2.1. Zebrafish Maintenance and Experimental Design

Adult 3-month-old female zebrafish (TU wild-type) were cultured in a semistatic system according to a previously established protocol [21]. Briefly, after acclimation for 2 weeks, zebrafish were exposed to varying concentrations of ENR (0.4, 4, and 40 μg/L) under two administration regimens: continuous exposure (Cont) and intermittent exposure (Int). ENR (CAS: 93106-60-6, purity ≥ 98%) was purchased from Aladdin Scientific (Shanghai, China). An ENR stocking solution was prepared in dimethyl sulfoxide (DMSO) and subsequently diluted to the nominal concentrations, ensuring a final DMSO concentration of 0.001% (*v*/*v*) across all treatments to maintain consistent solvent exposure. Zebrafish were maintained in aerated tap water (dissolved oxygen ~6 mg/L, pH 7.8–8.2, ammonia nitrogen ≤ 0.2 mg/L, nitrite ≤ 0.2 mg/L, temperature at 28 ± 0.5 °C) under a photoperiod of 14:10 h light/dark to ensure normal growth and baseline stability prior to exposure.

Each exposure group included three replicate tanks (*n* = 3), with each tank housing approximately 20 adult female zebrafish. To ensure sufficient tissue material for reliable detection while maintaining independent biological replicates, separate subsets of fish were randomly sampled for each downstream assay (biochemistry, qRT-PCR, Western blotting, histology, and lipidomics). For molecular and biochemical assays, livers from 3 randomly selected fish within a single replicate tank were pooled together to form one biological replicate. This strategy yielded 3 independent pooled samples per experimental group. All downstream statistical analyses were appropriately performed using these pooled samples as the independent biological units (*n* = 3). The exposure media were renewed every 48 h by replacing 50% of the tank water with freshly prepared ENR solution to maintain appropriate chemical concentrations [14]. In the Cont regimen, fish were exposed to ENR for 14 consecutive days. In the Int regimen, fish were exposed to ENR for 7 days, transferred to clean aerated tap water for a 5-day depuration phase, and then re-exposed to ENR for an additional 7 days, resulting in a total of 14 days of active ENR exposure. Both regimens were conducted simultaneously to ensure that the cumulative ENR exposure duration was identical. Following the exposure period, the zebrafish were euthanized following established animal welfare protocols. Adult zebrafish were deeply anesthetized using 150 mg/L Tricaine methanesulfonate (MS-222; buffered with sodium bicarbonate to pH 7.0–7.5). Following complete loss of equilibrium and cessation of opercular movement (approximately 3 min), euthanasia was ensured via rapid dissection and exsanguination to harvest target tissues. All animal procedures were conducted in strict accordance with the ethical guidelines of the Animal Research Usage Guide by Yunnan University.

### 2.2. Biochemical and Antioxidant Assays

Liver samples were homogenized in 9 volumes of ice-cold physiological saline (1:9, *w*/*v*) using a high-throughput tissue grinder, followed by centrifugation at 2500× *g* for 10 min at 4 °C. The resulting supernatant was collected for downstream assays (*n* = 3 pooled samples (each comprising 3 livers)). Hepatic levels of glucose (Glu), total cholesterol (TC), triglycerides (TG), alanine aminotransferase (ALT), aspartate aminotransferase (AST), catalase (CAT), superoxide dismutase (SOD), total antioxidant capacity (T-AOC), and malondialdehyde (MDA) were measured using commercial kits (Nanjing Jiancheng Bioengineering Institute, Nanjing, China) following the manufacturer’s instructions. Protein concentrations were determined using a BCA protein assay kit (Nanjing Jiancheng Bioengineering Institute, Nanjing, China), and all biochemical parameters were normalized to hepatic protein content.

### 2.3. Histopathological Analysis

For histological analysis, randomly selected zebrafish (*n* = 3 replicates per group) were fixed in 4% paraformaldehyde (Biosharp, Beijing, China) for 48 h. Fixed liver tissue was dehydrated through a graded ethanol series, cleared in methyl salicylate, and embedded in paraffin. Tissue sections (4 μm thickness) were prepared and stained with Periodic Acid-Schiff (PAS; Servicebio, Wuhan, China) for glycogen detection. Slides were examined under a CX20 light microscope (Sunny Optical Technology, Yuyao, China). Photomicrographs were captured and subsequently analyzed using Slide Viewer (v2.8.0), ImageJ (v1.54), and QuPath (v0.70) software.

### 2.4. Quantitative Real-Time PCR (qRT-PCR)

Total RNA was extracted from the liver tissues using the TRleasy™ Total RNA reagent (Yeasen, Shanghai, China) and homogenized with a high-throughput tissue grinder (*n* = 3 pooled samples (each comprising 3 livers)). RNA concentration and purity were assessed using a Nano Photometer NP80 Touch (Implen, Munich, Germany); only samples exhibiting A260/A280 ratios between 1.8 and 2.0 were utilized for downstream applications. Complementary DNA (cDNA) was synthesized using a reverse transcription kit (Yeasen, Shanghai, China). qRT-PCR was conducted on a real-time PCR system using UNICON^®^ Universal Blue qPCR SYBR Green Master Mix (Yeasen, Shanghai, China). The 10 μL reaction mixture contained 5 μL of 2× SYBR Green Master Mix, 0.2 μL of each forward and reverse primer (10 μM), 1 μL of 2-fold diluted cDNA, and 3.6 μL of RNase-free water. The thermocycling conditions consisted of an initial denaturation at 95 °C for 2 min, followed by 40 cycles of 95 °C for 10 s and 60 °C for 30 s, with fluorescence acquisition occurring during the annealing/extension step. Ribosomal protein l8 (*rpl8*) served as the reference gene, as its expression remained stable across all ENR exposure treatments. Relative gene expression was calculated using the 2^−ΔΔCt^ method [22]. All target genes and primer sequences used in this study are detailed in Supporting Tables S1 and S3.

### 2.5. Western Blot Analysis

Liver tissues were homogenized in RIPA lysis buffer (Servicebio, Wuhan, China) supplemented with a 50× protease inhibitor cocktail (Servicebio, Wuhan, China), phosphatase inhibitors, and PMSF at a ratio of 50:1:0.5:0.5 (*v*/*v*/*v*/*v*) (*n* = 3 pooled samples (each comprising 3 livers)). Homogenization was performed utilizing a high-throughput tissue grinder, followed by a 30-min incubation on ice and subsequent centrifugation at 12,000× *g* for 5 min at 4 °C. Equal amounts of protein were mixed with 2× SDS-PAGE loading buffer, denatured at 95 °C for 5 min, and separated via SDS-PAGE. The separated proteins were transferred onto activated PVDF membranes. These membranes were blocked with 5% non-fat milk in TBST for 2 h at room temperature, incubated with primary antibodies overnight at 4 °C, washed three times in TBST, and then incubated with HRP-conjugated secondary antibodies for 90 min at room temperature. Immunoreactive bands were visualized using BeyoECL Star (Beyotime, Shanghai, China) and imaged on a ChemiDoc system (Bio-Rad, Hercules, CA, USA). Band intensities were quantified using ImageJ (v1.54) software and normalized to GAPDH. Detailed antibody information and target protein are provided in Supporting Tables S2 and S3.

### 2.6. Liver Lipidomics

Untargeted lipidomic analysis was performed on three groups at the highest exposure concentration: the continuous-exposure group (Cont, 40 μg/L), the intermittent-exposure group (Int, 40 μg/L), and the control group (Ctrl) (*n* = 3 pooled samples (each comprising 3 livers)). Approximately 10 mg of liver tissue was homogenized in 200 μL of methanol/water (2:5, *v*/*v*) and 400 µL MTBE using a frozen tissue grinder (−10 °C, 50 Hz, 6 min), followed by low-temperature sonication for 30 min. After a 30-min incubation at −20 °C to precipitate proteins, samples were centrifuged (13,000× *g*, 4 °C, 15 min). The supernatant was evaporated to dryness under nitrogen. Dried extracts were reconstituted in 100 µL of isopropanol/acetonitrile (1:1, *v*/*v*), sonicated in an ice bath for 5 min, and centrifuged (13,000× *g*, 4 °C, 10 min) to yield the final supernatant for LC-MS analysis. Untargeted lipidomic profiling was performed on a Thermo UHPLC-Q Exactive HF-X system (Thermo Fisher Scientific, Waltham, MA, USA) equipped with an Accucore C30 column (100 × 2.1 mm, 2.6 µm) (Thermo Fisher Scientific, Waltham, MA, USA). Mobile phases consisted of (A) 10 mM ammonium acetate in 50% acetonitrile with 0.1% formic acid and (B) 2 mM ammonium acetate in acetonitrile/isopropanol/water [10:88:2, *v*/*v*/*v*] containing 0.02% formic acid. The flow rate was 0.40 mL/min at a column temperature of 40 °C. Mass spectra were acquired in both positive and negative electrospray ionization (ESI) modes. Data-dependent acquisition (DDA) was applied using normalized collision energies of 20, 40, and 60 V. Raw data were imported into LipidSearch (Thermo Fisher Scientific, Waltham, MA, USA) for baseline filtering, peak alignment, and lipid identification to generate the final data matrix. To ensure the stability and reproducibility of the analytical system, quality control (QC) samples were prepared by pooling equal volumes of the lipid extracts from all experimental samples. During the LC-MS/MS acquisition, one QC sample was injected after every 10 experimental samples to continuously monitor instrument performance. Following initial peak extraction, the data matrix underwent rigorous preprocessing. Variables were first filtered retaining only those lipid features containing non-zero values in at least 80% of the samples within any single experimental group. Any remaining missing values were subsequently imputed using the minimum value of the original data matrix. To minimize technical variance introduced by minor instrument fluctuations, the peak response intensities were normalized using the total sum normalization method. To ensure high data quality, variables exhibiting a relative standard deviation (RSD) > 30% across the QC samples were removed. Finally, to rigorously control for false positives during the identification of differential lipids, statistical significance was evaluated with corrections for multiple testing using the Benjamini–Hochberg False Discovery Rate (FDR).

### 2.7. Statistical Analysis

Values are presented as means ± standard error of the mean (SEM). Normality of data distribution was assessed using the Kolmogorov–Smirnov test, and homogeneity of variance was evaluated using Levene’s test. All datasets satisfied the assumptions for parametric testing. For comparisons involving three or more groups, significant differences were determined using one-way analysis of variance (ANOVA) followed by Tukey’s multiple comparison post hoc test. Differences between two independent groups were analyzed using an unpaired Student’s *t*-test. All statistical analyses were performed using SPSS 27.0 (IBM, Armonk, NY, USA). A *p*-value < 0.05 was considered statistically significant. Figures were prepared using GraphPad Prism 9 (GraphPad Software, San Diego, CA, USA).

## 3. Results

### 3.1. Hepatic Biochemistry and Antioxidant Responses Under Two Exposure Regimens

**Figure 1:**
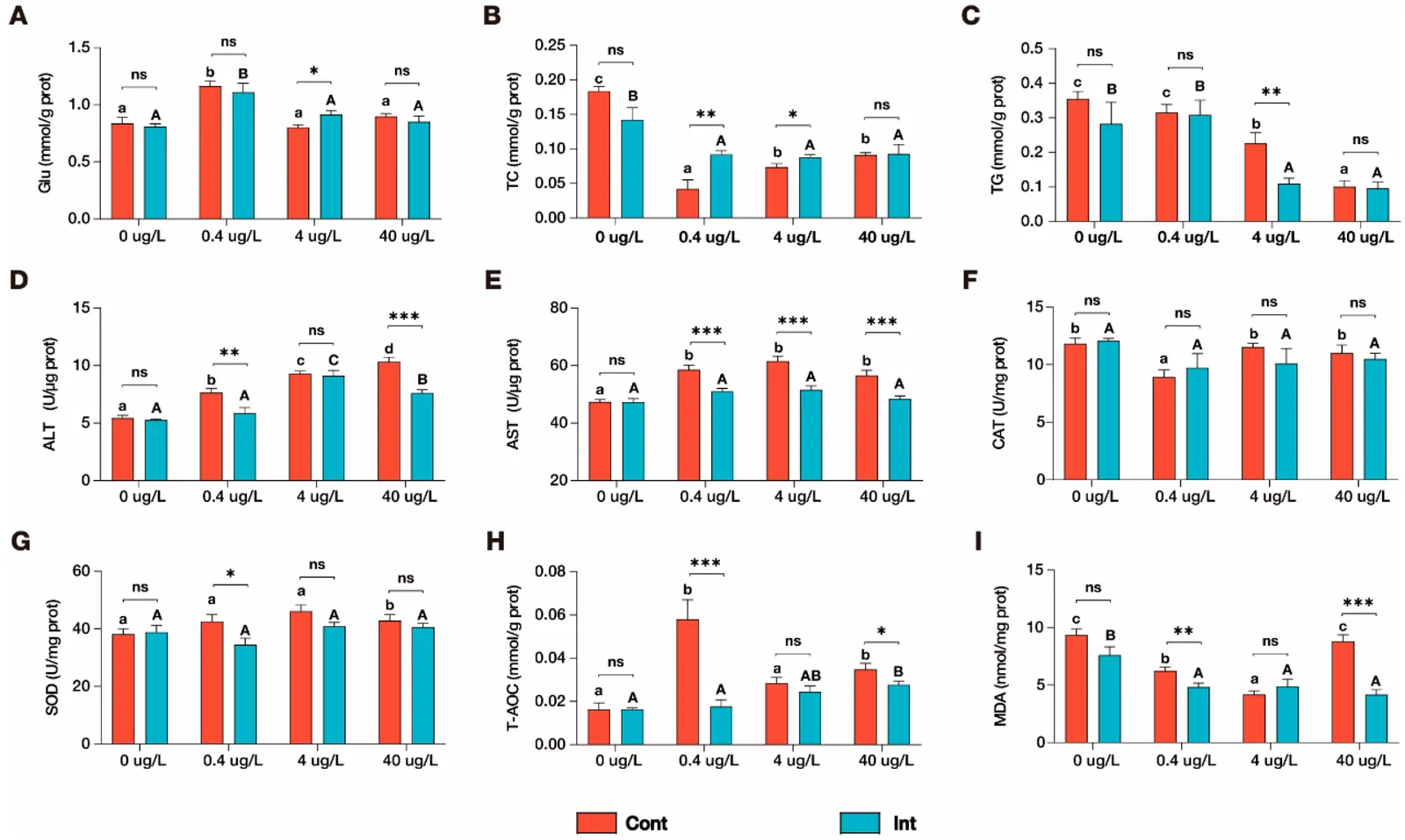
Basic hepatic biochemical parameters and antioxidant enzyme activities in zebrafish under two ENR exposure regimens. (**A**) Glucose (Glu), (**B**) total cholesterol (TC), (**C**) triglycerides (TG), (**D**) alanine aminotransferase (ALT), (**E**) aspartate aminotransferase (AST), (**F**) catalase (CAT), (**G**) superoxide dismutase (SOD), (**H**) total antioxidant capacity (T-AOC), and (**I**) malondialdehyde (MDA). Cont: continuous exposure groups; Int: intermittent exposure groups. Different lowercase and uppercase letters indicate significant differences among concentrations within the Cont and Int regimens, respectively (*p* < 0.05). Asterisks denote significant differences between the two regimens at the same exposure concentration (* *p* < 0.05, ** *p* < 0.01, and *** *p* < 0.001); “ns” indicates no significant difference (*p* > 0.05).

Regarding the hepatic biochemical and antioxidant indices, ENR exposure significantly altered multiple endpoints, with marked differences observed between the Cont and Int regimens (Figure 1). Notably, hepatic glucose (Glu) levels responded similarly in both regimens, being significantly elevated at 0.4 μg/L relative to the control (*p* < 0.05) and returning to baseline levels at the higher concentrations (Figure 1A). Furthermore, lipid metabolism parameters demonstrated higher sensitivity to ENR and displayed distinct concentration-dependent patterns. Hepatic total cholesterol (TC) was significantly depleted under both regimens compared with the control (Figure 1B). In the Cont regimen, TC sharply decreased at 0.4 μg/L and then slightly recovered at 4 and 40 μg/L, though it remained significantly below control levels. Conversely, the Int regimen caused a uniform reduction in TC across all exposure concentrations, with no significant dose-dependent variation among the exposure groups. Hepatic triglycerides (TG) remained unaffected at 0.4 μg/L in both regimens (Figure 1C). However, at 4 and 40 μg/L, TG levels decreased in a concentration-dependent manner under continuous exposure, whereas they remained uniformly suppressed without further dose-dependency under intermittent exposure.

In addition, hepatic transaminase activities were generally elevated by ENR. Alanine aminotransferase (ALT) activity increased in a strict concentration-dependent manner across all Cont groups (Figure 1D). Under the Int regimen, ALT significantly peaked at 4 μg/L and subsequently decreased at 40 μg/L, though it remained elevated compared with both the control and the 0.4 μg/L group. Notably, at 40 μg/L, ALT activity was significantly lower in the Int regimen than in the Cont regimen (*p* < 0.001). Aspartate aminotransferase (AST) activity was significantly elevated across all Cont groups with no dose-dependent variation, whereas it remained statistically unaffected under the Int regimen compared with the control (Figure 1E). Consequently, AST levels were significantly higher in the Cont regimen than in the matched Int regimen at all tested concentrations (*p* < 0.001).

The hepatic antioxidant system exhibited divergent responses between the two regimens. Catalase (CAT) activity was significantly suppressed at 0.4 μg/L under continuous exposure before returning to control levels at higher doses, whereas it remained unaltered under intermittent exposure (Figure 1F). Superoxide dismutase (SOD) activity remained largely stable across both regimens, showing no significant variation compared with the controls (Figure 1G). Total antioxidant capacity (T-AOC) exhibited a pronounced, transient spike at 0.4 μg/L under continuous exposure, whereas it demonstrated a gradual, concentration-dependent increase under the Int regimen, peaking significantly at 40 μg/L compared with the control (Figure 1H). Strikingly, hepatic malondialdehyde (MDA) content exhibited entirely disparate patterns between the regimens (Figure 1I). In the Cont groups, MDA displayed a U-shaped response, decreasing initially from the control to 4 μg/L before returning to baseline levels at 40 μg/L. In contrast, MDA levels under the Int regimen were significantly and uniformly reduced across all exposure groups.

### 3.2. Hepatic Glycogen Accumulation Changes Under Two Exposure Regimens

**Figure 2:**
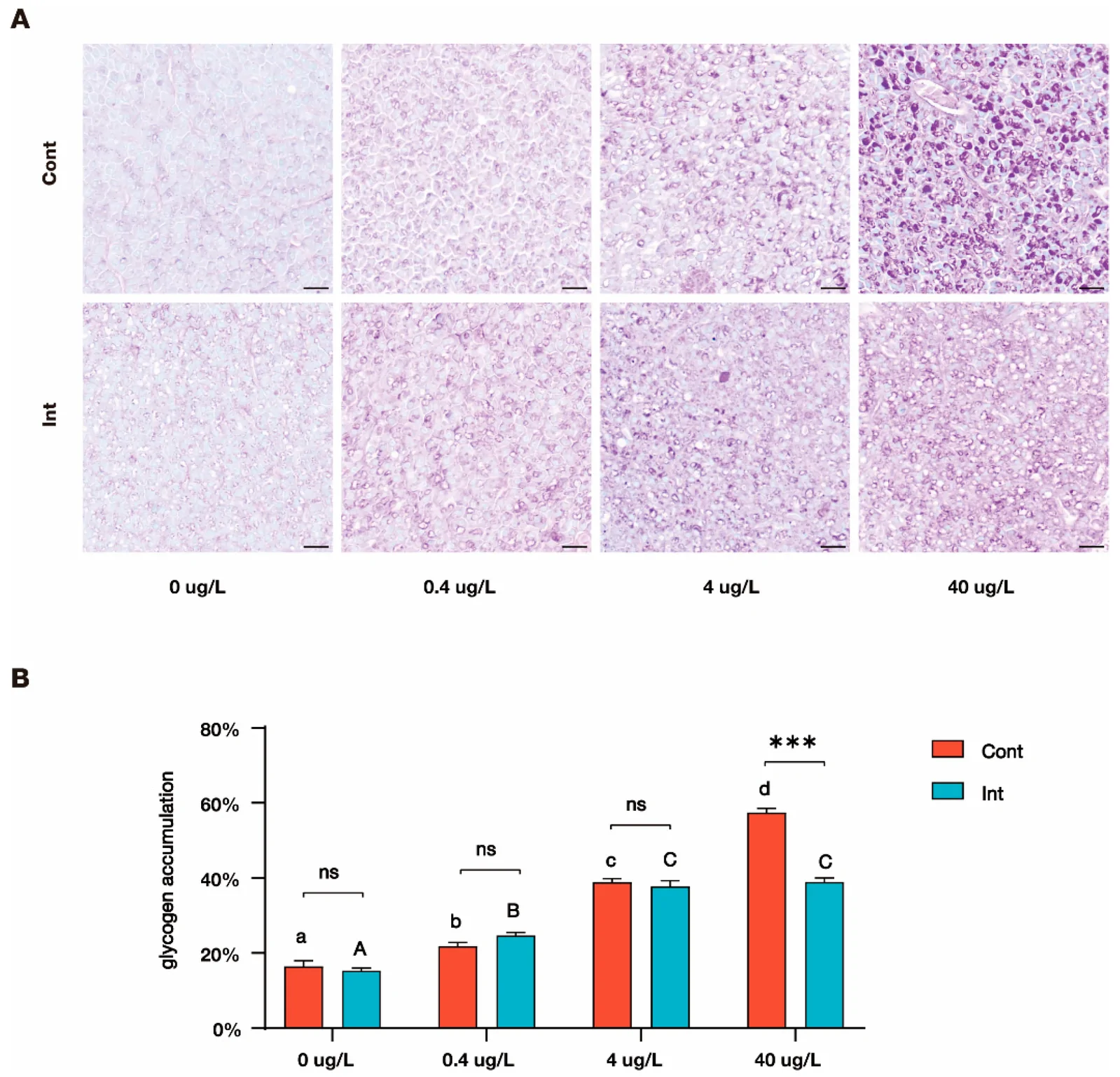
PAS staining of zebrafish liver tissue under two ENR exposure regimens. (**A**) Representative histological images of glycogen accumulation (indicated by dark purple/magenta granules) in zebrafish liver across different ENR exposure groups. Scale bar: 20 μm. (**B**) Quantification of hepatic glycogen accumulation. Cont: continuous exposure groups; Int: intermittent exposure groups. Different lowercase and uppercase letters indicate significant differences among concentrations within the Cont and Int regimens, respectively (*p* < 0.05). Asterisks denote significant differences between the two regimens at the same exposure concentration (*** *p* < 0.001); “ns” indicates no significant difference (*p* > 0.05).

PAS staining, which labels glycogen as magenta and nuclei as light blue, revealed marked differences in hepatic glycogen accumulation between the two ENR exposure regimens (Figure 2A). Under continuous exposure, a progressive concentration-dependent increase in hepatic glycogen density was observed across all ENR-treated groups. Conversely, under intermittent exposure, while hepatic glycogen content initially increased from the control to 4 μg/L, it plateaued, showing no further accumulation at 40 μg/L. Quantitative analysis confirmed these visual observations that glycogen accumulation increased in a strict concentration-dependent manner under the Cont regimen but saturated under the Int regimen (Figure 2B). Furthermore, at the highest exposure concentration (40 μg/L), the hepatic glycogen accumulation ratio was significantly higher in the continuous regimen compared with the intermittent regimen (*p* < 0.001).

### 3.3. Stress-Related Protein Expression Change Under Two Exposure Regimens

**Figure 3:**
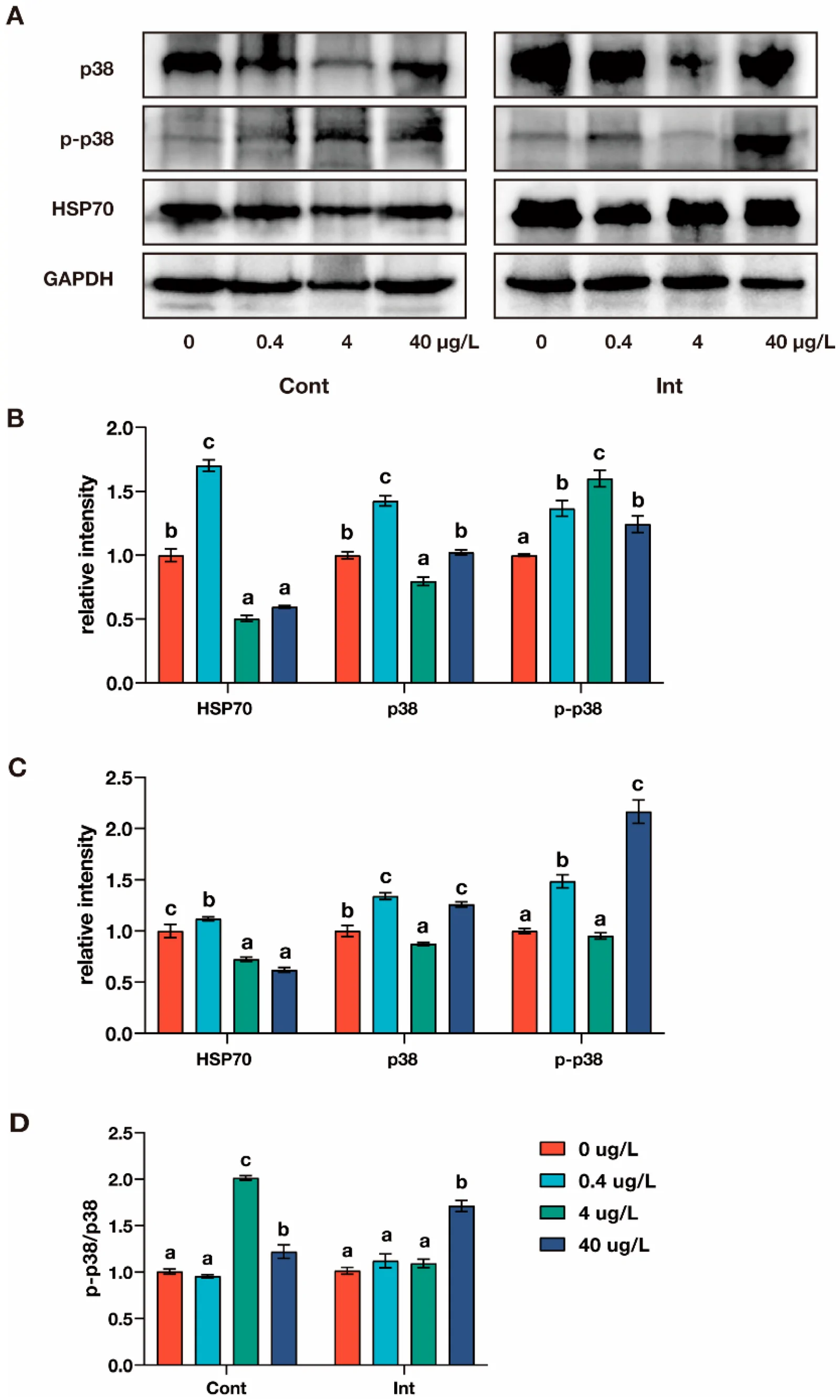
Stress and MAPK signaling pathway proteins change in zebrafish liver under two ENR exposure regimens. (**A**) Representative Western blot bands of HSP70, p38, p-p38, and GAPDH. (**B**) Relative protein expression levels (normalized to GAPDH) in the Int groups. (**C**) Relative protein expression levels (normalized to GAPDH) in the Cont groups. (**D**) The ratio of p-p38 to total p38 expression, indicating pathway activation. Cont: continuous exposure groups; Int: intermittent exposure groups. Different lowercase letters denote statistically significant differences among exposure concentrations within the same regimen (*p* < 0.05).

Western blot analysis was conducted to evaluate the hepatic expression of HSP70, p38, and phosphorylated p38 (p-p38) in zebrafish under the two ENR exposure regimens (Figure 3 and Figure S1). Under continuous exposure, HSP70 expression was significantly upregulated at 0.4 μg/L (*p* < 0.05) but markedly suppressed at 4 and 40 μg/L, falling well below baseline control levels (Figure 3A,B). Total p38 expression exhibited a similar biphasic pattern: it significantly increased at 0.4 μg/L, decreased at 4 μg/L, and returned to control levels at 40 μg/L (Figure 3B). Conversely, p-p38 levels increased in a strict concentration-dependent manner from 0.4 to 4 μg/L (*p* < 0.05). Although its expression slightly declined at 40 μg/L, p-p38 remained significantly elevated relative to the control across all ENR-treated groups (*p* < 0.05; Figure 3B).

Under intermittent exposure, HSP70 was significantly downregulated at 4 and 40 μg/L compared with the controls (*p* < 0.05; Figure 3A,C). The expressions of p38 and p-p38 displayed a consistent pattern: levels were significantly elevated above the controls at 0.4 and 40 μg/L (*p* < 0.05) but did not differ significantly from baseline at 4 μg/L (Figure 3B). To more accurately assess the activation of the p38 MAPK signaling pathway, the ratio of p-p38 to total p38 was calculated. Under continuous exposure, this ratio was significantly elevated at 4 μg/L (*p* < 0.05); while it decreased at 40 μg/L, it remained significantly above the control value (Figure 3D). In contrast, under intermittent exposure, the p-p38/p38 ratio was significantly elevated relative to the control only at the highest concentration of 40 μg/L (*p* < 0.05; Figure 3D).

### 3.4. Liver Lipid Profiling Change Under Two Exposure Regimens

**Figure 4:**
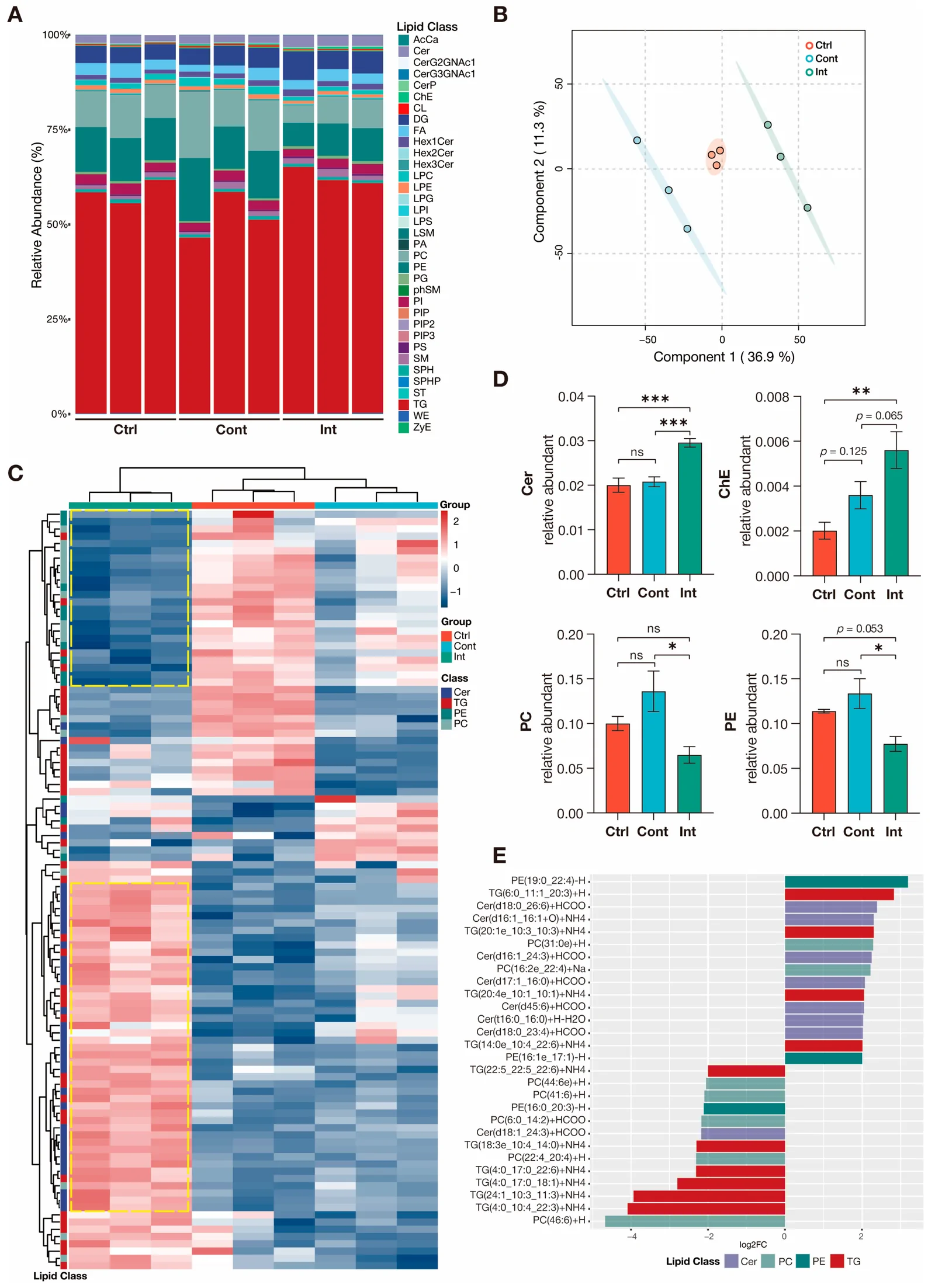
Hepatic lipidomic profiling of zebrafish under two ENR exposure regimens. (**A**) Relative abundance of major lipid classes across the control (Ctrl), continuous (Cont), and intermittent (Int) exposure groups (40 μg/L). (**B**) Principal component analysis (PCA) score plot of hepatic lipid profiles; shaded ellipses represent 95% confidence intervals. (**C**) Hierarchical clustering heatmap of all detected lipid species; yellow dashed boxes highlight distinct lipid clusters specifically altered under the Int regimen. (**D**) Relative abundances of ceramides (Cer), cholesteryl esters (ChE), phosphatidylcholines (PC), and phosphatidylethanolamines (PE). “ns” denotes no statistical significance; * *p* < 0.05, ** *p* < 0.01, and *** *p* < 0.001. (**E**) Top differentially abundant lipid species when comparing the Int regimen against the Cont regimen (|fold change| > 2, *p* < 0.05). Positive log2(Fold Change) values indicate significant upregulation in the Int group, whereas negative values indicate significant downregulation. Ctrl: control; Cont: continuous exposure at 40 μg/L; Int: intermittent exposure at 40 μg/L.

Untargeted lipidomic analysis was conducted on the control group (Ctrl) and the highest-concentration exposure groups (40 μg/L) for both the Cont and Int regimens to evaluate ENR-induced hepatic lipid remodeling. Across all three groups, triglycerides (TG), phosphatidylethanolamines (PE), and phosphatidylcholines (PC) constituted the most abundant lipid classes in the zebrafish liver (Figure 4A). Principal component analysis (PCA) demonstrated a complete separation of the 95% confidence intervals among the three groups. Notably, the two ENR-exposed groups diverged in opposite directions along Principal Component 1 relative to the control (Figure 4B), indicating that while both regimens substantially remodeled hepatic lipid metabolism, they did so via distinctly divergent pathways. Hierarchical clustering analysis further corroborated this divergence. The heatmap revealed specific clusters of extensively up- and downregulated lipid species unique to the Int regimen (highlighted by yellow dashed boxes), which were completely absent in the Cont group (Figure 4C).

Quantitative assessment of specific lipid classes demonstrated that the two regimens exerted opposing effects on major membrane phospholipids. Relative to the control, the relative abundances of PC and PE exhibited an upward trend under continuous exposure but a downward trend under intermittent exposure. Although neither trend reached statistical significance compared with the baseline (*p* > 0.05), these divergent trajectories resulted in significantly lower PC and PE levels in the Int group compared with the Cont group (*p* < 0.05; Figure 4D). Furthermore, ceramides (Cer) were significantly enriched in the Int group compared with both the Ctrl and Cont groups (*p* < 0.001; Figure 4D). Cholesteryl esters (ChE) were also significantly elevated in the Int group relative to the control (*p* < 0.01); however, the difference between the Int and Cont groups did not reach statistical significance (*p* = 0.065; Figure 4D). When comparing the most differentially abundant individual lipid species between the Int and Cont regimens (|fold change| > 2, *p* < 0.05), the upregulated profile in the Int group was dominated by specific ceramides (e.g., Cer(d18:0_26:6)) and long-chain triglycerides (e.g., TG(6:0_11:1_20:3)). Conversely, the downregulated profile was primarily comprised of distinct triglyceride species (e.g., TG(4:0_10:4_22:3)) and phosphatidylcholines (e.g., PC(46:6)) (Figure 4E).

### 3.5. Transcriptional Regulation of PC and PE Biosynthesis Under Two Regimens

**Figure 5:**
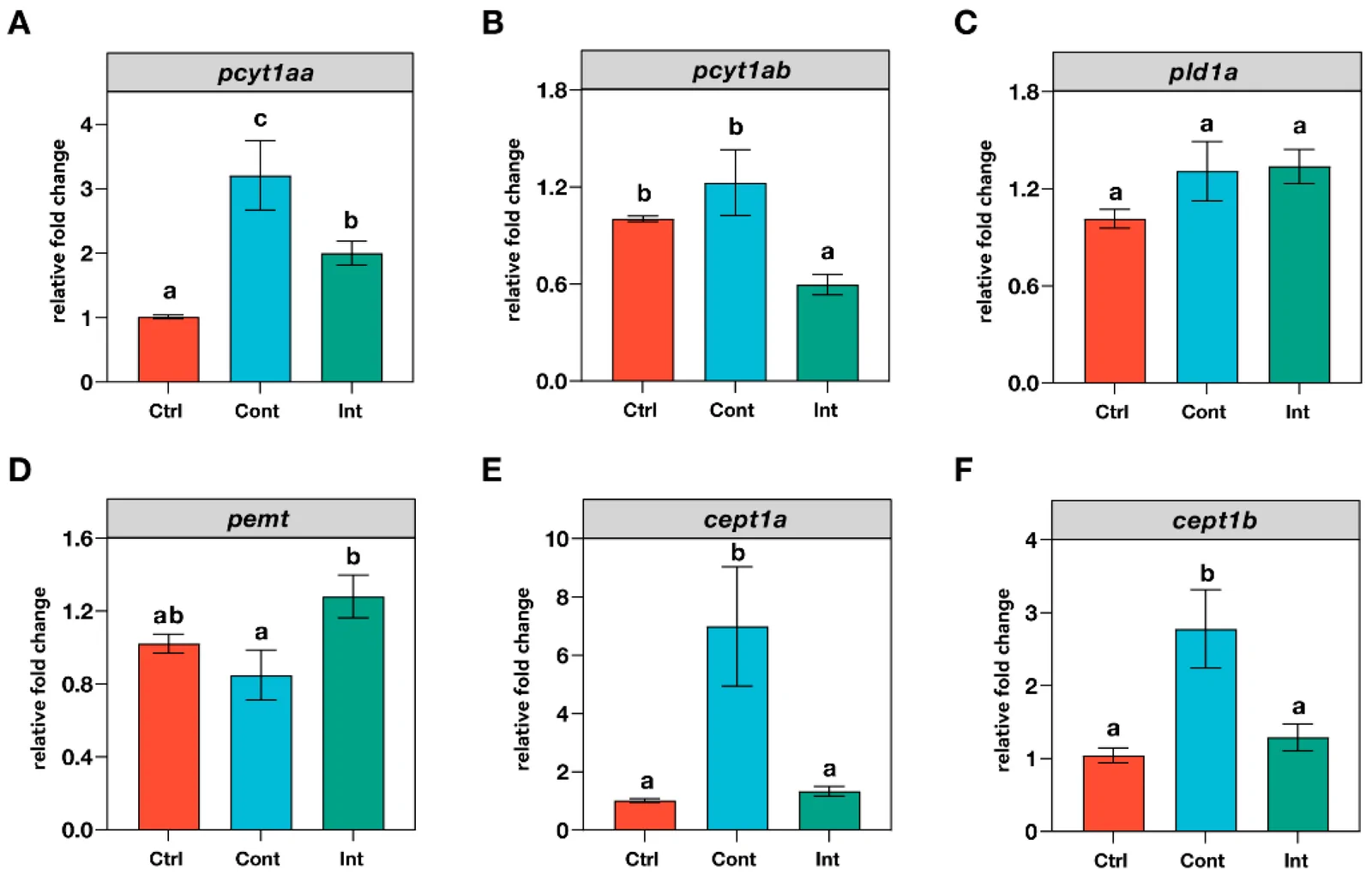
Transcriptional change of key genes involved in the de novo phosphatidylcholine (PC) and phosphatidylethanolamine (PE) biosynthesis pathways in zebrafish liver under two ENR exposure regimens. Analyses were performed at the highest exposure concentration (40 μg/L). Evaluated genes include (**A**) *pcyt1aa*, (**B**) *pcyt1ab*, (**C**) *pld1a*, (**D**) *pemt*, (**E**) *cept1a*, and (**F**) *cept1b*. Ctrl: control; Cont: continuous exposure at 40 μg/L; Int: intermittent exposure at 40 μg/L. Data are presented as relative fold changes normalized to the control. Different lowercase letters indicate statistically significant differences among the groups (*p* < 0.05).

To corroborate the lipidomic findings at the transcriptional level, alterations in the mRNA expression of key genes involved in the de novo biosynthesis pathways of PC and PE were determined, providing a mechanistic insight (Figure 5). This analysis was conducted on the control group and the highest exposure concentration (40 μg/L) for both the Cont and Int regimens.

Under the continuous regimen, the expression levels of *pcyt1aa*, *cept1a*, and *cept1b* were significantly upregulated compared with both the control and the Int group (*p* < 0.05; Figure 5A,E,F). The expression of *pcyt1ab* in the Cont group remained comparable to the control (*p* > 0.05; Figure 5B) but was significantly higher than that observed in the Int group. Conversely, the intermittent regimen induced a distinctly different transcriptional profile. While *pcyt1aa* was significantly upregulated relative to the control in the Int group, its expression remained significantly lower than that of the Cont group. Furthermore, *pcyt1ab* was significantly downregulated in the Int group compared with the baseline control (*p* < 0.05; Figure 5A). Notably, *pemt* exhibited significantly higher expression in the Int group relative to the Cont group (*p* < 0.05; Figure 5D), although neither exposure group differed significantly from the control. Finally, the expression of *pld1a* remained unaffected across all experimental groups, with no significant differences observed (*p* > 0.05; Figure 5C).

## 4. Discussion

### 4.1. Intermittent Exposure Mitigates ENR-Induced Hepatotoxicity and Oxidative Imbalance

The teleost liver serves as the primary hub for xenobiotic detoxification and energy homeostasis. Under toxicant challenge, organisms must actively redirect metabolic flux away from long-term storage toward the rapid energy production required for detoxification and cellular repair [23]. In the present study, both ENR exposure regimens induced pervasive reductions in hepatic TC and TG, reflecting a classical, stress-induced energy reallocation. Furthermore, Glu was significantly elevated at the lowest dose (0.4 μg/L) under both regimens before returning to baseline at higher concentrations. This initial spike likely represents an acute, hormetic energy-mobilization response to mild chemical stress, whereas normalization at higher doses signals either short-term metabolic adaptation or the exhaustion of accessible glycogen reserves [24].

Despite this shared baseline metabolic reallocation, the severity of hepatocellular injury was profoundly dictated by the temporal exposure regimen. Hepatic transaminases are essential clinical biomarkers for hepatocellular membrane integrity [25]. Continuous exposure triggered a strict, concentration-dependent elevation of ALT and a pervasive upregulation of AST, confirming progressive and substantive liver damage [26]. In stark contrast, intermittent exposure completely protected against AST elevation and significantly reduced ALT leakage at the highest concentration (40 μg/L). These data unequivocally demonstrate that, under an identical cumulative ENR dose, the continuous regimen exacerbates hepatotoxicity, whereas the intermittent regimen’s depuration phase significantly mitigates cellular injury and preserves membrane permeability [27].

This structural protection is mechanistically linked to divergent oxidative trajectories. Xenobiotic exposure typically triggers excessive ROS generation, which can disrupt calcium homeostasis, deplete ATP, and directly induce hepatocyte toxicity [28]. Interestingly, the primary enzymatic antioxidants (SOD and CAT) remained largely unperturbed across both regimens, contrasting with earlier findings in ENR-exposed large yellow croaker [20] and florfenicol-exposed zebrafish [29]. This discrepancy suggests that, at these environmentally realistic concentrations, zebrafish mount a robust compensatory defense primarily through the modulation of total antioxidant capacity (T-AOC) [30,31].

Under continuous exposure, an initial acute spike in T-AOC at 0.4 μg/L collapsed at higher concentrations, leading to unchecked ROS accumulation and a severe, U-shaped rebound in MDA, a key biomarker of lipid peroxidation [32,33]. Conversely, the intermittent regimen facilitated a sustainable, concentration-dependent enhancement of T-AOC. This persistent defense successfully neutralized ENR-induced ROS, maintaining MDA at uniformly suppressed levels across all concentrations [34]. Ultimately, the continuous regimen breached the liver’s oxidative threshold, leading to the upregulation of MDA at 40 μg/L whereas the intermittent depuration phase provided a critical physiological buffering window that prevented oxidative collapse.

### 4.2. ENR-Induced Compensatory Hepatic Glycogen Storage

Histopathological evaluations provided direct visual corroboration of these biochemical findings. Previous studies have identified that antibiotic exposure frequently induces inflammatory infiltration, cellular swelling, and lipid degeneration in the teleost liver [19,35,36]. Beyond these structural lesions, the present findings revealed that ENR exposure profoundly disrupts hepatic glycogen storage. As the principal storage form of glucose, glycogen is rapidly mobilized to maintain systemic energy homeostasis [37,38]. PAS staining demonstrated that both administration modes induced excessive hepatic glycogen accumulation, albeit with distinct dynamic trajectories. Continuous exposure drove a strict, concentration-dependent surge in glycogen content. In contrast, while intermittent exposure initially elevated glycogen levels, this accumulation rapidly plateaued between 4 and 40 μg/L, resulting in a markedly lower glycogen burden at the highest dose compared with the continuous group.

This pronounced glycogen deposition—coinciding with severe TC and TG depletion—likely reflects a compensatory metabolic shift toward glycogenesis, triggered when normal lipid storage and mobilization pathways are severely impaired by chemical stress or diet style [39,40]. Pathological hepatic glycogen accumulation is a hallmark of metabolic dysregulation, known to interfere with carbohydrate absorption and ultimately compromise overall fish growth and fitness [41]. Collectively, these findings underscore that continuous ENR exposure exacerbates glycolipid dysregulation, inflicting composite functional and structural damage to the zebrafish liver.

### 4.3. Regimen-Dependent Thresholds for MAPK Activation and Cellular Stress

At the molecular level, cellular fate under xenobiotic challenge is governed by the coordinated interaction of molecular chaperone systems and signal transduction networks [42]. Heat shock protein 70 (HSP70), a highly conserved early stress biomarker, plays a pivotal role in maintaining protein-folding homeostasis [43,44]. Under continuous exposure, HSP70 displayed an inverted U-shaped dose–response curve significantly upregulated at 0.4 μg/L, to mitigate ROS-induced damage [45], but profoundly suppressed at higher doses. This biphasic pattern aligns with previous reports indicating that high toxicant concentrations suppress chaperone expression due to severe cellular damage and widespread translational impairment [46]. Notably, under intermittent exposure, HSP70 was also significantly reduced at intermediate and high doses. Given the overall tissue protection observed under this regimen, this downregulation likely reflects a strategic metabolic reprioritization: the depuration phase lowers the physiological baseline requirement for continuous, energy-costly chaperone overexpression.

The transition from adaptive oxidative stress to pathological inflammation and apoptosis is critically dependent on the mitogen-activated protein kinase (MAPK) signaling cascade [47]. Because HSP70 acts as an upstream modulator of p38 MAPK, their coordinated upregulation initiates robust cellular defenses [48]. Under continuous exposure, total p38 expression mirrored the biphasic trend of HSP70. However, the ratio of phosphorylated p38 to total p38 (p-p38/p38) remained significantly elevated across both intermediate and high concentrations. This sustained p38 activation drives downstream inflammatory and apoptotic pathways, thereby accelerating pathological outcomes under continuous chemical pressure [49].

Conversely, under intermittent exposure, the p-p38/p38 ratio was significantly elevated only at the highest concentration (40 μg/L). Crucially, this robust p38 reactivation coincided with significantly suppressed MDA levels, suggesting that it represents a successful, adaptive signaling response rather than unmitigated oxidative injury. A key finding of this study is that the two administration regimens dictate distinctly different MAPK activation thresholds: continuous exposure triggered pathological pathway activation at just 4 μg/L, whereas the intermittent regimen buffered this stress, requiring a tenfold increase to 40 μg/L to achieve significant activation.

### 4.4. Adaptive Lipid Remodeling and Compensatory Phospholipid Biosynthesis

Because MAPK signaling heavily influences hepatic lipid metabolism [50], combing the changing of hepatic lipid level and the compensatory hepatic glycogen storage, we performed untargeted lipidomic profiling to delineate the effects of the temporal exposure regimen. The hepatic lipid profiles of the two ENR-exposed groups clustered completely apart from both the control and each other, with phosphatidylcholines (PC) and phosphatidylethanolamines (PE) displaying diametrically opposed trajectories. PC and PE increased under continuous exposure but decreased under intermittent exposure. This aligns with previous work demonstrating that short-term ENR exposure induces severe metabolic disturbances in marine medaka (*Oryzias melastigma*), including significant elevations in hepatic PC and PE [51].

Organisms modulate PC/PE synthesis pathways to maintain membrane homeostasis under external stress. The Kennedy pathway enzymes, such as phosphocholine cytidylyltransferase (pcyt1aa/ab) and choline/ethanolamine phosphotransferase (cept1a/b), are rate-limiting for de novo PC biosynthesis [52]. Under continuous exposure, the significant upregulation of *pcyt1aa*, *cept1a*, and *cept1b* indicates strong activation of this de novo pathway, accounting for the elevated PC and PE pools [53,54]. Intermittent exposure, however, attenuated this broad de novo activation. Instead, it triggered a unique, significant upregulation of phosphatidylethanolamine N-methyltransferase (pemt), which catalyzes the sequential methylation of PE to PC [55]. This suggests a strategic metabolic shift: intermittent exposure redirects PC synthesis toward the PE-methylation pathway, utilizing it as a compensatory route to maintain essential PC supplies when cyclical recovery phases dial down de novo synthesis.

Furthermore, the intermittent regimen uniquely induced a highly significant accumulation of ceramides (Cer). While excessive ceramide classically impairs mitochondrial oxidative respiration and promotes lipid peroxidation [56,57,58], the present biochemical data explicitly demonstrated that the intermittent group successfully suppressed MDA accumulation. This implies that under intermittent conditions, ceramide primarily functions in cellular signaling rather than pathological membrane destruction. Importantly, hepatocellular ceramide is a potent upstream activator of the MAPK cascade, capable of catalyzing extensive p38 phosphorylation [59,60]. This mechanistic link perfectly corroborates the Western blot findings, where the elevated p-p38/p38 ratio at 40 μg/L directly coincided with Cer enrichment. Thus, ceramide-driven MAPK activation under the intermittent regimen represents a precisely regulated, adaptive signaling response facilitated by the depuration phase, contrasting sharply with the progressive hepatotoxicity driven by continuous exposure.

### 4.5. Limitations of This Study

While this study elucidates the divergent hepatotoxic trajectories of continuous and intermittent ENR exposure, certain methodological limitations must be acknowledged. To eliminate baseline physiological variance, our experimental model was restricted to adult female zebrafish; consequently, because toxicological responses and lipid metabolism are highly sex-dependent, these results may not directly translate to males or other life stages. Furthermore, to secure sufficient tissue mass for reliable omics and biochemical analyses, tissues from three individual fish were pooled to generate a single biological replicate. While technically necessary, this pooling strategy inherently minimizes inter-individual biological variability and may mask individual differences in the response to ENR exposure. Additionally, although our water renewal protocol was based on established literature that satisfies OECD testing guidelines, direct analytical measurement of the tank water would have provided a more accurate verification of the nominal exposure concentrations. Furthermore, our mechanistic conclusions rely heavily on transcriptomic data to corroborate the phenotypic lipidomic shifts. Since mRNA abundance is not a definitive proxy for functional protein levels or enzymatic activity, further targeted proteomic and functional validations of these signaling pathways are warranted.

## 5. Conclusions

Taken together, these findings underscore the importance of temporal exposure patterns as a critical determinant of ENR hepatotoxicity in zebrafish. Continuous ENR exposure overwhelmed hepatic antioxidant capacity, drove progressive hepatocellular injury, and triggered pathological p38 MAPK activation alongside severe disruptions to lipid and glycogen homeostasis. Conversely, intermittent exposure provided a critical physiological buffering window. This recovery period significantly mitigated structural liver damage—evidenced by a 26.3% reduction in ALT activity relative to the continuous regimen—facilitated adaptive antioxidant responses and initiated compensatory lipid remodeling. Overall, the present findings provide a basis for challenging the traditional dose-centric paradigm in toxicological risk assessment. For the aquaculture industry, these results provide implications for optimizing antibiotic administration modes to significantly alleviate hepatic stress in farmed fish, thereby promoting animal welfare and advancing sustainable antibiotic stewardship.

## Data Availability

The data presented in this study are available upon request from the corresponding author.

## Supplementary Materials

The following supporting information can be downloaded at: https://www.mdpi.com/article/10.3390/biology15161354/s1, Table S1. Primer sequences used for qRT-PCR in this study; Table S2. Antibodies used for Western blot in this study; Table S3. Abbreviations in this study; Figure S1. Western blot images in this study.
