# Supplementary material for: Intermittent Administration Alleviates Enrofloxacin-Induced Hepatotoxicity in Zebrafish via Adaptive Lipid Remodeling and p38 MAPK Signaling
Source: Biology (Basel). 2026 Aug 10;15(16):1354. doi: 10.3390/biology15161354 (PMC13509458; doi:10.3390/biology15161354)
Supplement: Supplementary file 1 [file biology-15-01354-s001.zip › biology-4461132-supplementary.pdf]

Supporting Table S1. Primer sequences used for qRT-PCR in this study.

Gene name	Primer sequence (5′→3′)	Accession No.	Amplicon size (bp)
rpl8	F: TGCAAACAGAGCCGTTGTTG	NM_200713.1	78
	R: TAAGCACGTCCTGCCTTCAG		
pcyt1aa	F: AAGCACACGGAGCCTGAAT	NM_001017634.1	260
	R: GATCCCATCAGCGTACACCC		
pcyt1ab	F: CACGCGAGTTCATCGGAAAC	NM_001020735.1	118
	R: CTGGGACTCTGTCTGGGACT		
pemt	F: GAGTCGCTGCTGGACAGAAA	NM_200158.1	226
	R: CACCAAACAGACGGGTCAGA		
cept1a	F: AGCTGAAGAGGCTGGAGGA	NM_001144792.3	239
	R: CAAACCCAAAGCACATGCCA		
cept1b	F: GTTCTTCTGCTGTTTCGCCG	NM_001109717.1	179
	R: TTACGGGAATCGGAGACTGC		
pld1a	F: CTCAGGTCGAAACGTCACCA	NM_001160095.1	211
	R: CGAGGGCATGAGGTTTTCCT		

Supporting Table S2. Antibodies used for western blot in this study.

Name	Dilution	Manufacturer	Catalog No.
GAPDH Monoclonal antibody	1:50 000	Proteintech, Wuhan, China	60004-1-1g
Recombinant Anti-HSP70 antibody (Mouse mAb)	1:1 000	Servicebio, Wuhan, China	GB15241
Recombinant Anti-p38 antibody (Rabbit mAb)	1:1 000	Servicebio, Wuhan, China	GB154685
Recombinant Anti-Phospho-p38 (T180/Y182) antibody (Rabbit mAb)	1:1 000	Servicebio, Wuhan, China	GB153380
HRP-conjugated Affinipure Goat Anti-Mouse IgG (H+L)	1:5 000	Proteintech, Wuhan, China	SA00001-1
Multi-rAb HRP-Goat Anti-Rabbit Recombinant Secondary Antibody (H+L)	1:5 000	Proteintech, Wuhan, China	RGAR001

Supporting Table S3. Abbreviations in this study.

Abbreviation	Full name
ENR	Enrofloxacin
Glu	Glucose
TG	Triglyceride
TC	Total Cholesterol
SOD	Superoxide Dismutase
CAT	Catalase
T-AOC	Total Antioxidant Capacity
MDA	Malondialdehyde
ALT	Alanine aminotransferase
AST	Aspartate aminotransferase
ROS	Reactive oxygen species
Ctrl	Control Group
Cont	Continuous exposure
Int	Intermittent exposure
PAS	Periodic Acid-Schiff staning
GAPDH	Glyceraldehyde 3-phosphate dehydrogenase
HSP70	Heat shock protein 70
p38	p38 mitogen-activated protein kinase
pp38	Phospho-p38 mitogen-activated protein kinase
TNFα	Tumor necrosis factor-alpha
PE	Phosphatidylethanolamine
PC	Phosphatidylcholine
Cer	Ceramide
ChE	Cholesteryl ester
rpl8	Ribosomal protein L8
pcyt1aa	Phosphate cytidylyltransferase 1A, choline a
pcyt1ab	Phosphate cytidylyltransferase 1A, choline b
pld1a	Phospholipase D1a
pemt	Phosphatidylethanolamine N-methyltransferase
cept1a	Choline/ethanolamine phosphotransferase 1a
cept1b	Choline/ethanolamine phosphotransferase 1b

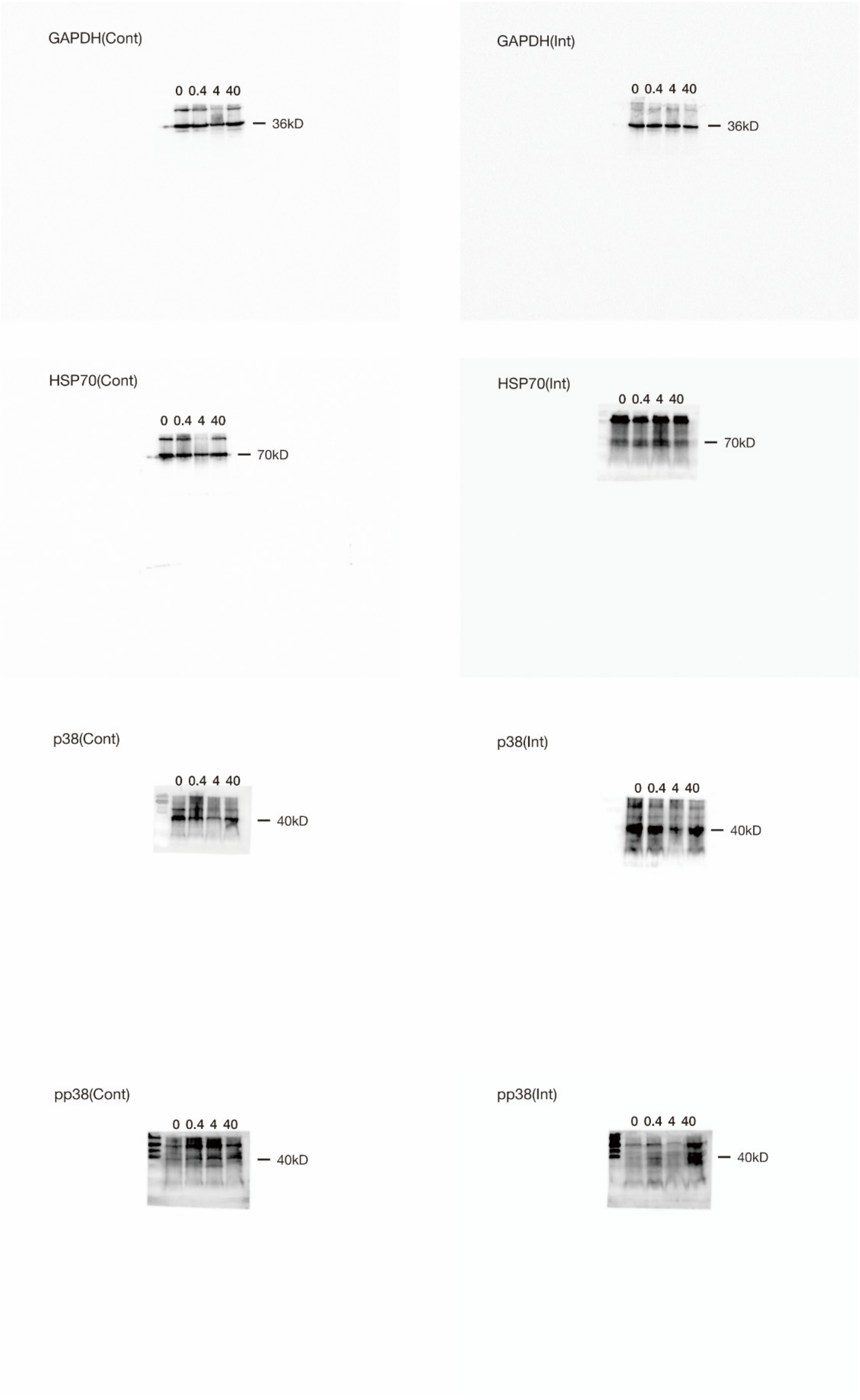

Supporting Figure S1. Western blot images in this study.
